# New Evidence on the Effect of Medical Insurance on the Obesity Risk of Rural Residents: Findings from the China Health and Nutrition Survey (CHNS, 2004–2011)

**DOI:** 10.3390/ijerph15020383

**Published:** 2018-02-23

**Authors:** Jian Zhao, Chang Su, Huijun Wang, Zhihong Wang, Bing Zhang

**Affiliations:** National Institute for Nutrition and Health, Chinese Center for Disease Control and Prevention, Beijing 100050, China; zhaojian131023@163.com (J.Z.); suchang@ninh.chinacdc.cn (C.S.); wanghj@ninh.chinacdc.cn (H.W.); wangzh@ninh.chinacdc.cn (Z.W.)

**Keywords:** new rural cooperative medical insurance, general obesity, abdominal obesity, moral hazard, China

## Abstract

The obesity rate in China has risen significantly in the past few decades. While a number of causes for the rise in obesity have been explored, little attention has been paid to the role of health insurance per se. This study aims to investigate the impact of health insurance on the risk of obesity in rural China using longitudinal data from the China Health and Nutrition Survey (CHNS). We employed pooled ordinary least squares (OLS), probit estimation, and pooled two-stage least squares (2SLS) for an instrumental variable (IV). The IV model revealed that New rural cooperative medical insurance (NRCMS) participation had a significant positive impact on people’s tendency towards unhealthy lifestyles, for instances, high-fat food (8.01% for female and 7.35% for male), cigarette smoking (25% for male), heavy drinking (25% for female), sedentary activity (6.48 h/w for female and 6.48 h/w for male), waist circumference (1.97 cm for female and 1.80 cm for male), body mass index (0.58 kg/m^2^ for female), which in turn leads to an elevated probability of general obesity (51% for female) and abdominal obesity (24% for female and 20% for male). An “ex ante moral hazard” is prevalent in rural China, which should not be ignored by policymakers so as to minimize the related low efficiency in the process of promoting the universal coverage of insurance.

## 1. Introduction

Obesity is a serious public health problem that needs to be addressed urgently both in developed and developing countries [1,2,3,4]. Along with economic development and social changes, the epidemic of obesity is rapidly growing among men and women in China [5]. According to the Chinese Nutrition and Chronic Disease Survey report in 2015, the obesity rates among men and women were 12.1% and 14.4%, respectively. Moreover, excessive body weight is an important risk factor for mortality and morbidity from cardiovascular diseases, type-2 diabetes, musculoskeletal disorders, and cancers, causing nearly 3 million deaths annually worldwide [6,7,8]. There is a myriad of studies that have tried to address the role of insurance in obesity rates over the past decades. While there is no argument that genetic predisposition plays an important role in obesity, the increase in obesity is mostly attributed to lifestyle choices and environmental factors, including a high-fat food dietary preference and a shift toward a sedentary lifestyle, and sex may play a role in such an association [9]. These lifestyle choices may be driven in part by the moral hazard effect.

Economic theories have indicated that an insurance-related ex ante moral hazard causes a reduction in self-protection behaviors and in turn leads to obesity [10,11]. While expanding universal health insurance coverage has been the primary health policy in health care reform around the world in recent years, the question of whether health insurance matters for health has long been a central issue for debate. Previous studies have suggested that rural residents are more often overweight and obese than urban residents [12,13,14,15]. Although those living outside of urban areas tend to be of lower socioeconomic position [16,17], rurality increases the risk of being overweight or obese independent of compositional factors, such as age, education, income, and marital status [13,14]. This suggests that the rural context has an important role to play in obesity development. In the rural areas of China, the rapid increase of the New Rural Cooperative Medical Insurance (NRCMS) coverage rate is certainly striking. The key issue, however, is whether it would result in an improvement in the health of its participants.

China’s health care system did not match its rapid economic development during the same period. To bridge this gap, the Chinese government has substantially reformed its health care system in recent years [18,19]. The goal is to provide universal coverage for basic health care to every Chinese citizen. However, it has been shown that health insurance could have negative outcomes and can lead to deadweight loss in social welfare. Rural residents make up almost half of China’s total population. Mortality in the rural population is also substantially higher than that in the urban population. In addition, a higher proportion of rural residents are below the average poverty levels compared with urban residents. Therefore, it is meaningful to study the effect of the NRCMS on the health of Chinese rural residents.

Evidence addressing the causal effect of insurance on the risk of obesity in rural areas is sparse, so more study is warranted. The main challenge for evaluating the effects of health insurance on obesity risk is the endogeneity problem caused by simultaneity and omitted variables. Endogeneity refers to the fact that an explanatory variable is correlated with unobservable heterogeneity that is relegated to an error term. Several studies to date have adopted different econometric approaches to deal with these issues. Courbage and Coulon used an instrumental variable (IV) approach to explore the effect of as one method for controlling the unobservable heterogeneity that jointly determines health insurance and health behavior [20]. Anderson used data from the U.S. Panel Study of Income Dynamics (1991–2003) to estimate a structural model and found that health insurance has significant incentive effects of unhealthy lifestyle choices, all of which have been shown by the medical literature to cause serious long-term health damage [21].

This study attempts to fill the theoretical and practical gap by exploring the effects of the NRCMS scheme on the risk of obesity in rural China while correcting for underlying endogeneity. Our instrument for a respondent’s NRCMS health insurance status is the data of county-level NRCMS inception, an instrument used in the previous literature to estimate the impact of NRCMS on health status and the utilization of preventive care [22]. We attempt to isolate the effects of an “ex ante moral hazard”, where people with insurance may change their lifestyle choices towards weight control. This study utilizes the China Health and Nutrition Survey (CHNS, 2004–2011), a nation-wide longitudinal survey including the whole period of the NRCMS, to analyze the impact of the NRCMS. With the advantage of updated and comprehensive data, our findings may also be meaningful to other developing countries facing similar challenges on the way to establishing universal health insurance coverage.

## 2. Subjects and Methods

### 2.1. Study Population

We used the data of the China Health and Nutrition Survey (CHNS) for the present investigation, which was designed to examine how the social and economic transformation in China has affected the health and nutritional status of the Chinese population [23]. The CHNS is an ongoing prospective study across 239 communities within 9 out of all 31 provinces that vary in demography, geography, economic development, and public resources in mainland China (Guangxi, Guizhou, Heilongjiang, Henan, Hubei, Hunan, Jiangsu, Liaoning, and Shandong). Two cities and four counties were randomly selected based on a multistage cluster random sampling scheme [24] in each of the provinces, and the sampling scheme is reported in detail elsewhere [5]. The present analysis was based on four rounds of CHNS data (2004, 2006, 2009, and 2011). Our final sample consisted of 19,577 person-year observations (holding hukou and living in rural areas) aged 18–65 years with complete data on demographics, socioeconomic status, insurance status information, and 3-day, 24-h dietary recalls in a survey year. Among them, 9845 were insured and 9732 were uninsured. We excluded participants who were pregnant, lactating, and who had implausible energy intakes (<600 kcal or >4000 kcal) from the analysis.

This study was approved by the institutional review boards of the University of North Carolina at Chapel Hill and the National Institute for Nutrition and Health, Chinese Center for Disease Control and Prevention. Written informed consent was provided by each participant (2015017).

### 2.2. Health Insurance Data

The key explanatory variable in our analysis is a dummy indicating whether an individual had NRCMS coverage in the survey year. According to the CHNS questionnaire, if a respondent answered “Yes” to the coverage of NRCMS and lived in a community with NRCMS, then she is defined as an NRCMS enrollee for our analysis. This variable can change over time.

### 2.3. Lifestyle Choices

Drinking and smoking habits are two dummy variables representing whether the respondent had a habit of drinking spirits and smoking cigarettes during the past year, respectively. Measurements of sedentary leisure time among adults were only available from 2004 in the CHNS study. The sedentary activity is calculated by the average time (in hours) spent on five activities (watching television, internet browsing, online chatting, playing board games, and reading newspaper/magazines) per week.

### 2.4. Dietary Data

Dietary intake at the individual level was assessed by using three consecutive 24-h dietary recalls in each wave of the CHNS [25]. The participants were asked to report the kinds and amounts of the food and beverage items (measured in g) they consumed at home and away from home during a 24-h period [26]. Based on the Chinese Food Composition Table, we measured nutrition intake data by (1) the respondent’s daily calorie intake per day during a 3-day measurement period and (2) the dietary structure as indicated by the percentage of fat, carbohydrates, and protein in the respondent’s daily calorie intake [27].

### 2.5. Anthropometrics and Obesity Indicators

The body weight and height of each participant were measured by well-trained health workers following standardized procedures (SECA 880 scales and a SECA 206 wall-mounted metal tape). The body mass index (BMI) was calculated by dividing the weight (kg) by the square of height (m^2^) of each participant [28,29]. In accordance with the National Health and Family Planning Commission of the People’s Republic of China, the participates were defined as having general obesity if the BMI ≥28.0 kg/m^2^ [29]. Waist circumstance (WC) was measured from the midpoint between the lower border of the rib cage and the iliac crest to the nearest 0.1 cm. Abdominal obesity was defined as a waist circumference ≥85 cm for women and ≥90 cm for men, according to a new definition that was published by the national health and family planning commission in 2013 for Chinese adults.

### 2.6. Social-Demographic Data

For our analysis, the major demographic characteristics are age (years) and gender. We categorize gender as male or female. Socioeconomic variables include marital status, education level, income level, and employment status. Marital status is categorized as married and single (including never married, divorced, widowed, and separated). Employment status is divided into employed and unemployed. Education level reflects the years of formal schooling. Educational attainment was classified as: primary/illiterate, junior school, and high school/above. The per-capital annual income in each survey was inflated to values in 2011 by adjusting for the consumer price index and then categorizing it into tertiles as low, medium, and high level [30].

## 3. Statistical Analysis

Statistical analyses were performed using STATA 13.0 (STATA, Stata Corp, College Station, TX, USA). The values were reported as means and standard errors for continuous variables or as proportions of the total for categorical variables. We subdivided data according to different demographic characteristics. Among all the variables, we adjusted age, sedentary activity time, daily calorie intake, the share of calories from carbohydrate, and the share of calories from fat as continuous variables and gender, drinking, smoking, education level, marital status, employment status, general obesity, and abdominal obesity as dummy variables.

To investigate the effects of NRCMS on lifestyle behaviors, we first employed a pooled ordinary least square (OLS) model covering the whole period 2004–2011. The equation we used for this model is

*y_ipt_* = *α*_0_ + *α*_1_*NRCMS_ipt_* + *α*_2_*X_ipt_* + *δ_t_* + *α_p_* + *ε_ipt_* (1)

where *y_ipt_* denotes the heath behavior of individual *i* in province *p* and year *t*. This includes such outcome variables as sedentary time, daily calorie intake, and the proportion of carbohydrates, protein, and fat in total calorie intake. *NRCMS_ipt_* is a dummy variable (taking on a value 0 or 1) indicating whether the individual is enrolled in NRCMS in year *t*; *X_ipt_* contains a set of control variables including age, education level, income level, marital status, and employment status; *δ_t_* indicates time fixed-effects that control for the unobservable characteristics that are constant across all regions; α*_p_* indicates province fixed-effects that control for unobservable characteristics of a province that are constant over time; and *ε_ipt_* is the random error that varies with individual, province, and year. The parameter *α*_1_ indicates the impact of the NRCMS on the participant’s health behaviors, thus it is the coefficient of interest.

Where our dependent variables were binary, we also estimated a probit model and report the average marginal effects. This includes such outcome variables as whether the respondent currently drinks spirits, smokes, has general obesity, or has abdominal obesity. This is to characterize the potentially non-linear impact of insurance and to avoid the problem of natural heteroscedasticity with OLS. The probit model takes on the following form:

*y*′*_ipt_* = *α*_0_ + *α*_1_*NRCMS_ipt_* + *α*_2_*X_ipt_* + *δ_t_* + *α_p_* + *ε_ipt_* (2)

P = (y_ipt_ = |NRCMS, X, δ, α) = P (y ′_ipt =_ |NRCMS, X, δ, α)                    = *G* (*α*_1_*NRCMS_ipt_* + *α*_2_*X_ipt_* + *δ_t_* + *α_p_* + *ε_ipt_*) (3)

where *y_ipt_* is a dummy indicating whether individual *i* in province *p* and year *t* smokes, drinks, is sedentary, has general obesity, or has abdominal obesity. *y′_ipt_* is the latent variable specifying the tendency of such behaviors. If *y′_ipt_ >* 0, then *y_ipt_* = 1; otherwise *y_ipt_* = 0. *G* is the cumulative distribution function of random error *ε_ipt_.* Unlike the OLS model in Equation (1), the *α*_1_ in the probit model only indicates the impact of NRCMS coverage on the tendency of risk-taking (*y′_ipt_*). To obtain the (non-linear) marginal effect of NRCMS on the observed probability of risking-taking behaviors, we use the sample average of individual marginal effects calculated by a finite-difference method.

Note that in Equations (1)–(3), the OLS and probit of *α*_1_ and *α*_2_ are still heavily biased because of endogeneity, that is, a correlation between insurance status and the error, *α*_1_.

The problem of adverse selection in the schemes creates a higher likelihood of correlation between insurance participation and the error term, which induces a bias in the coefficient of health insurance on the health behavior equation. This condition leads to a positive association between insurance status and risk of obesity, because higher health risk people are more likely to enroll in an insurance plan than others.

To address this endogeneity of NRCMS participation, we use the IV method in this paper. We constructed pooled two-stage least squares (2SLS) to calculate IV estimates for insurance status.

NRCMS*_ipt_* = β + β_1_ Country*_ipt_* +β_2_X*_ipt_* +γ_t_ + η*_p_* + θ*_ipt_* (4)

y*_ipt_* = α_0_ + α_1_NRCMS” *_ipt_* + α_2_X*_ipt_* + δ*_t_* + α*_p_* + ε*_ipt_* (5)

There are two requirements for this instrument to be valid. The first requirement is the existence of a high correlation between whether an individual is participating in an NRCMS (*NRCMS_ipt_*) and whether his/her county of residence has implemented the NRCMS. The second requirement is that it should not directly impact the individual’s health-related behaviors (*y_ipt_*) independent of *NRCMS_ipt_.* We use whether the individual’s county of residence has implemented NRCMS in the survey year (*Country_ipt_*) as the IV for individual insurance participation. As China’s NRCMS campaign was implemented at the county level after its initiation in 2003, and rural residents could only join the NRCMS after the programme was adopted in her county of residence, there should be a strong correlation between individual participation and county enrollment. In addition, the timing of a county’s NRCMS implementation is centrally planned by the Ministry of Health of China and provincial health bureaus; thus, it is not likely to directly influence the individual health behavior independent of the insurance channel.

The Hausman test was conducted to measure the exogenous status of insurance. The power conditions of the IV are formally tested using the *F* values for the significance of *β*_1_ in the first-stage regressions. Moreover, we use the Basmann test of over-identification restrictions to make sure our approach and instruments are valid.

## 4. Results

### 4.1. Sample Description

**Figure 1:**
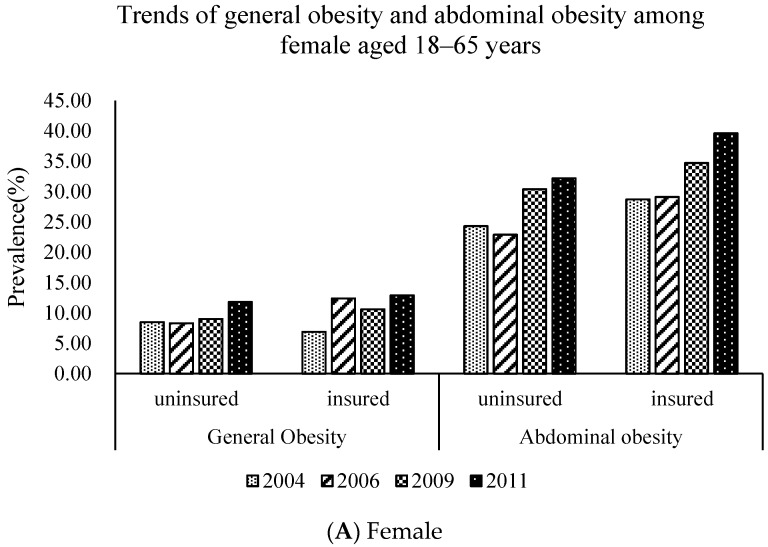

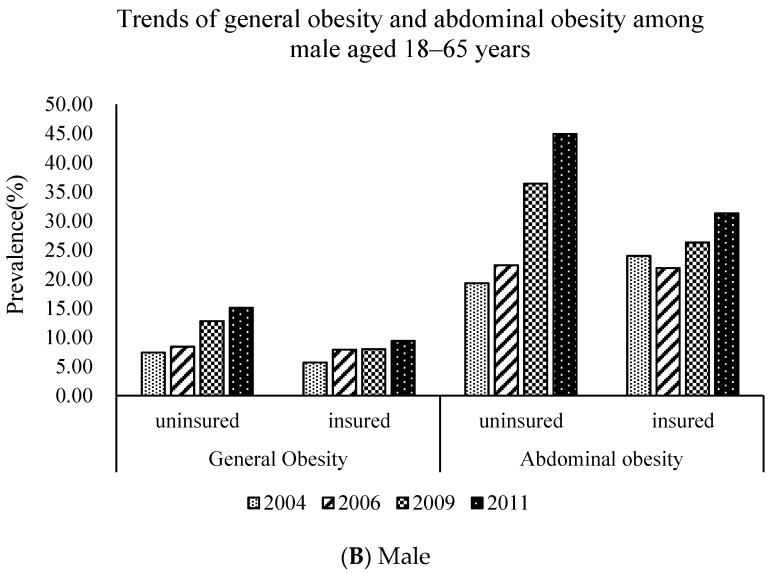
Trend of general obesity and abdominal obesity in Chinese rural residents (Female (**A**) and Male (**B**)) by insurance status from 2004 to 2011.

Descriptive differences between the insured and uninsured group in different genders are presented in Table 1. Several statistically significant differences between the two subgroups were observed. For instance, the average age and proportion of primary/illiterate education, low-income group, married status, employed, cigarette smoking, and high-fat food diet preferencing insured females were higher than those of uninsured females (*p* < 0.001). Similar patterns appear in males. It is noteworthy the BMI and WC of the female insured group were significantly higher than those of the uninsured group (*p* < 0.05). Moreover, general obesity and abdominal obesity are increasing significantly in both the insured and uninsured groups from 2004 to 2011 (Figure 1). NRCMS participation increased the likelihood of having general obesity by 12% and abdominal obesity by 35% for females (*p* < 0.05).

**Figure 2:**
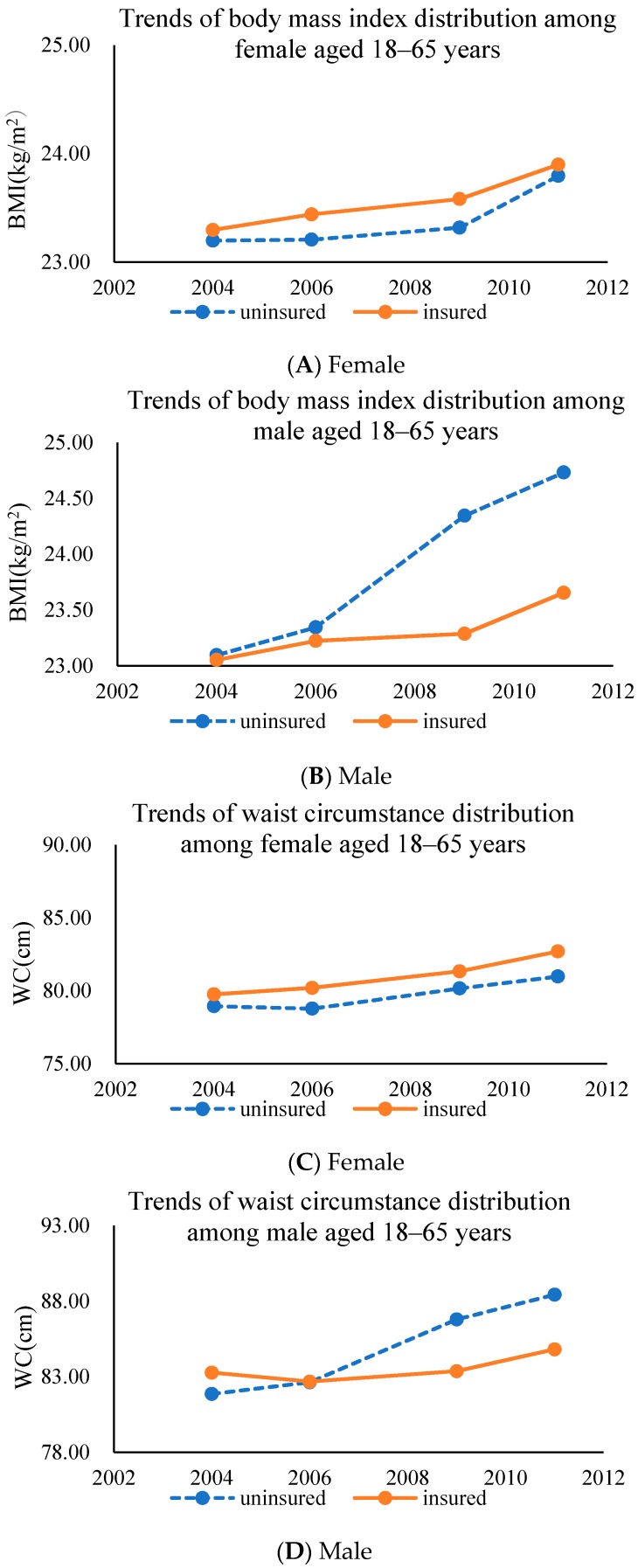
Shift in distribution of Body Mass Index (BMI) and Waist Circumstance (WC) in Chinese rural residents ((**A**) BMI of Female, (**B**) BMI of Male, (**C**) WC of Female, (**D**) WC of Male) by insurance status from 1991 to 2011.

Table 2 describes the trends of the NRCMS participation in different gender groups from 2004 to 2011. There were significant temporal trends in social demographic characteristics (age, education levels, income levels, marital status, and employment status), lifestyle behaviors (smoking, drinking, sedentary activity, and a high-fat food dietary preference) and obesity indicators (WC, BMI, general obesity, and abdominal obesity). Specifically, for females, the insured size was 247 in 2004, 1042 in 2006, 2011 in 2009, and 1947 in 2011. In addition, the insured size was 229 in 2004, 924 in 2006, 1813 in 2009, and 1632 in 2011 for males. The average age of the participants increased from 44.2 to 46.6 years for females, and 44.6 to 46.1 years for males (*p* < 0.05). The proportion of primary/illiterate education increased to 35% for females (*p* < 0.05), while participants with high school/above education decreased to 15% for males (*p* < 0.01). The uninsured can be considered more socio-economically deprived in many respects. For example, the proportion of the high-income group increased significantly in the whole population, among which females increased to 37% and males increased to 52% (*p* < 0.01). The smoking rate of female participants increased to 4% (*p* < 0.05). Sedentary activity time decreased to 16.1 h/w for females and almost 17.0 h/w for males (*p* < 0.05). In addition, by 2011, the share of calories from fat among females and males increased to 31.9% and 31.0%, respectively, and the share of calories from carbohydrate decreased to 56.1% and 55.4%, respectively (*p* < 0.01). The BMI of the insured increased to 23.7 kg/m^2^ and 23.9 kg/m^2^ for males and females, respectively (*p* < 0.05). Meanwhile, the WC increased to 82.7 cm and 84.8 cm for males and females, respectively. (*p* < 0.01). Despite the increasing trend in all of the survey years, the BMI and WC of females in the insured group was higher than those of females in the uninsured group, which is opposite to that of men (Figure 2). Furthermore, by 2011, the average general obesity prevalence rate of females increased to 13.0%. The abdominal obesity prevalence rate increased to 31% and 40% for males and females, respectively.

### 4.2. Impact Estimation Results

Table 3 reports the main empirical results in different gender groups. Ordinary least squares (OLS) (Model 1) and the probit model (Model 2) provide baseline estimates. These are compared with a two-stage least squares (2SLS) (Model 3) instrumental variable (IV) estimation that directly address the endogeneity of insurance with health conditions. We have found differences among these models reflecting the extent to which the estimation models can deal with the endogeneity problem. For instance, among females, we found that the OLS estimation (Model1) coefficients for daily calorie intake, the share of calories from carbohydrate, the share of calories from fat, and sedentary activity time, 0.03, 0.65, −0.18, and −0.35, respectively, differed significantly from the IV coefficients (Model 3) of −0.01 for daily calorie intake, −9.00 for the share of calories from carbohydrate, 8.01 for the share of calories from fat, and 6.48 for sedentary activity time.

As Model 3 showed, NRCMS participation increased the likelihood of a high-calorie food preference by 8.01% for females and 7.35% for males, and participation increased the likelihood of a low-carbohydrate diet by 9.00% for females and 9.74% for males. It also has a statistically significant positive impact on people’s tendency towards cigarette smoking (25% for males), heavy drinking (25% for females), sedentary activity time (6.48 h/w and 5.47 h/w for females and males, respectively), WC (1.97 cm and 1.80 for females and males, respectively), and BMI (0.58 kg/m^2^ for females). In addition, NRCMS participation increased the likelihood of having general obesity by 53% for females, and participation increased the likelihood of having abdominal obesity by 30% and 27% for females and males, respectively. After controlling for the lifestyle behaviors in Table 4, NRCMS participation increased the likelihood of increasing waist circumstance by 1.51 cm, increasing BMI by 0.47 kg/m^2^, and having general obesity by 4% for females. NRCMS participation increased the likelihood of having general obesity by 51% for females and having abdominal obesity by 21% for males.

With regard to other covariates, age was found to have a positive effect on the probability of females having unhealthy lifestyle choices, such as smoking, drinking, consuming a larger share of calories from fat, having general obesity, and having abdominal obesity (Appendix A Table A3). Females with high school/above education had almost a 100 kcal decrease in calorie intake and reduced their likelihood of abdominal obesity by 22%. In addition, females with a medium or higher income can reduce their likelihood of abdominal obesity by 8% and 11%, respectively. In addition, employed females have a relatively healthy dietary energy source; they consume a 2.7% larger share of carbohydrates and a 2.6% smaller share of fat. Meanwhile, they trend to spend less time on sedentary activities and are less likely to have general obesity and abdominal obesity. As shown in Appendix A Table A4, age was found to have a positive effect on the probability of males making unhealthy lifestyle choices, such as smoking, drinking, and consuming a larger share of calories from fat, and therefore have 3% and 5% more probability of having general obesity and abdominal obesity, respectively. Males with a junior education level consume a larger share of calories from fat and trend to spend about 2 more hours on sedentary activities per week. Moreover, males with a tertiary education reduced total calorie intake by 96 kcal, and are 5% more likely to consume a larger share of calories from fat. They were more associated with drinking and sedentary activities and an increase in the probability of abdominal obesity by 2%. Married males are 20% more likely to drink spirits, they spend 1.9 less hours on sedentary activities, and reduce their probability of smoking by 10%. They are 25% more likely to have general obesity.

We ran the Hausman test and the F-test on the instruments and the Basman test for over-identification restrictions. All these tests suggest that our approach and instruments are valid. (Appendix A Table A3, Table A4 and Table A5).

## 5. Discussion

Currently, obesity is widespread worldwide. The latest data on the prevalence of overweight and obese adults in 20 European countries shows that more than half of the European population is overweight and obese [31]. In China, the prevalence of overweight and obese adults has increased rapidly due to large shifts in dietary and lifestyle factors [32], in which there are considerable regional and gender differences due to regional disparities in social and economic development and gender disparities in physiological and lifestyle factors, etc. [33]. The gender-specific characteristics of populations at high risk of developing obesity should be taken into consideration when designing interventional programs [34]. Despite more than a century of research, new health risks and adverse effects have frustrated our ability to produce lasting cost-effective results. This paper explores the impact of insurance itself on lifestyle behaviors and risk of obesity in different gender groups. Using updated longitudinal data, our study provided new evidence on the effect of the NRCMS on health conditions of rural Chinese.

The expansion of health insurance is a popular public issue in China, and the Chinese government has made great efforts to launch public health programs for various populations to improve the accessibility, affordability, and quality of health care. However, health insurance is not without costs and the ex ante moral hazard problems (changing health-related behaviors) associated with insurance add to these costs. Some domestic and international preliminary explorations have linked insurance with obesity risk from the view of identifying ex ante moral hazards, but the results are inconsistent. For example, Jay Bhattacharya used data from the Rand Health Insurance experiment and found that being insured increased BMI and obesity [35]. Ir Kelly and S. Markowitz used data from the Behavioral Risk Factor Surveillance System to determine the potential effect of having health insurance on measures of body weight and found that having insurance is associated with a higher body mass but not a higher probability of being obese [10], consistent with the conclusion of kenkel and card [36]. Xue Zheng Q. used data from the China health and nutrition survey and found that NRCMS participation increased unhealthy lifestyles that in turn lead to a risk of being overweight, indicating that an insurance-induced ex ante moral hazard is present in rural China [37].

In the present study, OLS, Probit, and IV estimation are conducted to fully investigate how the NRCMS affects the lifestyle behaviors of its participants, such as dietary preference, smoking, drinking, sedentary activities, and obesity-related conditions. The sub-sample results of our study reflect noticeable gender differences in health-related behavior and obesity conditions. Besides this, our study shows that insurance coverage provides a significant incentive to consume a larger proportion of daily calories from fat but a smaller proportion from carbohydrates. Supportive evidence can be found in, for example, Shuang M., who studied the effect of the NRCMS on rural household food consumption and found that insurance participants have higher calorie, carbohydrate, fat, and protein intakes than those who are uninsured [38]. Due to a high-calorie diet having long been considered a risk factor for being overweight or obese or having other chronic diseases, unhealthy eating habits once again show that the participation in the NRCMS may cause problems with efforts to reduce the number of self-protected people in the rural population.

The result on the positive relationship between insurance participation and the tendency for females to have an alcohol problem indicates that the NRCMS has gender-specific influences on people’s tendency towards heavy drinking. Meanwhile, the harmful consequences of alcohol consumption are well-documented. At a personal level, alcohol consumption is causally related to cardiovascular diseases, various cancers, liver diseases, and psychiatric ailments [39]. Additionally, it is worth noting that women’s alcohol consumption may potentially affect the growth of children in a family, domestic violence, and economic instability. Some previous research, such as Andersen, found that joining catastrophic public insurance programs increases the likelihood of a person’s drinking alcohol. Our results suggest that NRCMS coverage leads to an increase in smoking behavior of males in rural China.

In addition, our results indicate that the coverage of the NRCMS can increase the intensity of sedentary activity, which in turn leads to higher risks of chronic diseases. This finding is also supported by Dave and Kaestner who found that receiving insurance has positive impacts on drinking and negative impacts on exercise, which is generally consistent with an increase in unhealthy behavior [11]. Furthermore, the results reveal the positive effect of the NRCMS on general obesity (females) and abdominal obesity (males), once again proving the existence of an ex ante moral hazard in the process of NRCMS universal coverage. Our results show that the NRCMS increases the body mass index and waist circumstance. Supportive evidence can be found in, for example, Jay Bhattacharya, who used data from the Rand Health Insurance experiment and found strong evidence that being insured increases body mass index and obesity.

The present study has many strengths. First, we used updated longitudinal data to observe the dynamic aspects of NRCMS coverage with other control variables. It provides new evidence to improve reforms of the medical and health system and the integration of rural insurance systems. Second, we provide evidence of gender-specific characteristics in exploring the impact of insurance on the risk of developing obesity. Third, the sample size is large with a wide age range, and the staff were trained in the study’s methodology and standardization in different parameters at the same time by the same scientists. In addition to baseline OLS estimation and the Probit model, IV estimation modeling corrects the omitted variable bias and increases the accuracy of the estimates.

While this study provides critical insight into health insurance, it nevertheless has several limitations. First, the CHNS does not present national data, and the vast western areas (north (Heilongjiang and Liaoning), the central areas (Shandong, Jiangsu, and Henan), and the south areas (Hubei, Hunan, Guangxi, and Guizhou) of China were not included in the present study. Second, dietary data were collected using three consecutive 24-h dietary recalls, which might show relatively limited variations for a subject compared to nonconsecutive 24-h recalls. However, the average intake over 3 days can offer a relatively valid estimate of nutrient intake, as shown in an earlier study using the CHNS data [40].

## 6. Conclusions

This study fills a theoretical and practical gap by exploring whether the expansion in the NRCMS induces ex ante moral hazard among its enrollees. Specially, we utilize the national data from the CHNS to analyze the impact of NRCMS participation on individual health-related behaviors. New and stronger evidence that having NRCMS affects the unhealthy lifestyles in turn leads to an elevated probability of having general obesity for females and abdominal obesity for males. In the process of promoting the universal coverage of insurance, the phenomenon of an “ex ante moral hazard”, which is prevalent in rural areas in China, should not be ignored by policymakers so as to minimize the related low efficiency. We call for further reforms in health insurance to reduce the negative outcomes and loss in social welfare. For example, the government can adjust nutrition policies while reforming the medical system. Second, health insurance prices can adjust insurance premiums based on an individual’s lifestyle behaviors (such as healthy eating). Third, the government-sponsored health insurance scheme can work with private insurance companies to offer incentive programs, such as rebates to individuals who participate in insurance-sponsored healthful activities. Fourth, to strengthen health awareness, there is a need to educate rural Chinese rural adults to choose a relatively healthy lifestyle and reduce the risk of obesity. However, further research is needed to determine if these activities represent a move toward efficiency in the health insurance market.

## Figures and Tables

**Table 1:** Basic characteristics of the health insurance status in different gender groups.

Characteristic	Female			Male		
	Total	Uninsured	Insured	Total	Uninsured	Insured
Age (year)	44.86 (11.63)	43.95 (11.65)	45.70 (11.55) ***	44.34 (12.14)	43.72 (11.97)	44.96 (12.27) ***
Primary/illiterate	0.50 (0.50)	0.42 (0.49)	0.58 (0.49) *	0.32 (0.47)	0.28 (0.45)	0.37 (0.48)
Junior school	0.34 (0.47)	0.34 (0.47)	0.340 (0.47) **	0.42 (0.49)	0.38 (0.49)	0.47 (0.50)
High school/above	0.16 (0.37)	0.24 (0.43)	0.10 (0.28)	0.25 (0.43)	0.34 (0.48)	0.16 (0.36) ***
Income (Low)	0.39 (0.49)	0.44 (0.50)	0.34 (0.48) ***	0.28 (0.45)	0.30 (0.46)	0.25 (0.43) ***
Income (Medium)	0.36 (0.48)	0.33 (0.48)	0.38 (0.49) ***	0.32 (0.47)	0.32 (0.47)	0.32 (0.47)
Income (High)	0.25 (0.43)	0.23 (0.42)	0.28 (0.45) ***	0.40 (0.49)	0.38 (0.49)	0.43 (0.50) ***
Married	0.91 (0.28)	0.90 (0.30)	0.93 (0.26) ***	0.89 (0.32)	0.87 (0.33)	0.90 (0.30) ***
Employed	0.65 (0.47)	0.61 (0.49)	0.69 (0.46) ***	0.84 (0.37)	0.81 (0.39)	0.87 (0.34) ***
Smoking	0.04 (0.19)	0.03 (0.16)	0.04 (0.21) ***	0.65 (0.48)	0.64 (0.48)	0.66 (0.47) **
Drinking	0.08 (0.27)	0.08 (0.21)	0.09 (0.28)	0.67 (0.47)	0.65 (0.48)	0.70 (0.46) **
Sedentary activity (h/w)	16.34 (13.64)	16.53 (13.96)	16.15 (13.32)	18.28 (15.11)	19.23 (15.79)	17.31 (14.32) ***
Total Calorie (1k kcal)	2.11 (0.60)	2.11 (0.60)	2.10 (0.60)	2.56 (0.77)	2.52 (0.75)	2.60 (0.78) ***
Fat (%E)	29.18 (11.26)	28.23 (11.60)	30.10 (10.84) ***	28.8 (10.98)	28.02 (11.29)	29.55 (10.61) ***
Carbohydrates (%E)	57.96 (11.71)	58.87 (12.05)	57.08 (11.30) ***	56.95 (12.29)	57.84 (12.50)	56.04 (12.01) ***
WC (cm)	80.45 (10.23)	79.29 (9.73)	81.55 (10.57) **	83.69 (10.11)	83.63 (10.03)	83.74 (10.18)
BMI (kg/m^2^)	23.51 (3.61)	23.29 (3.56)	23.72 (3.66) **	23.47 (3.48)	23.55 (3.38)	23.39 (3.58) **
General Obesity	0.10 (0.30)	0.09 (0.28)	0.12 (0.32) ***	0.09 (0.29)	0.09 (0.29)	0.08 (0.28) *
Abdominal Obesity	0.30 (0.46)	0.26 (0.44)	0.35 (0.48) ***	0.26 (0.44)	0.26 (0.44)	0.27 (0.44)
Sample Size	10,305	5058	5247	9272	4674	4598

Note: (1) The data come from the China Health and Nutrition Survey (CHNS) longitudinal data, 2004, 2006, 2009, and 2011. (2) The statistics reported are the sample mean with standard deviation in parenthesis. (3) ***, **, and * indicate statistical significance at the 1%, 5%, and 10% level, respectively. (4) %E, share of calories; WC, waist circumstance; BMI, body-mass index.

**Table 2:** Basic characteristics of trends of NRCMS participation in different gender groups from 2004 to 2011.

Characteristic	Female				Male			
	2004	2006	2009	2011	2004	2006	2009	2011
Age (year)	44.15 (0.67)	45.47 (0.33)	45.13 (0.27)	46.61 (0.26) ***	44.62 (0.01)	45.00 (0.01)	43.98 (0.01)	46.09 (0.01) ***
Primary/illiterate	0.64 (0.03)	0.60 (0.02)	0.57 (0.01)	0.56 (0.01) *	0.34 (0.01)	0.36 (0.01)	0.38 (0.01)	0.38 (0.01)
Junior	0.27 (0.03)	0.32 (0.01)	0.35 (0.01)	0.35 (0.01) **	0.46 (0.03)	0.44 (0.02)	0.49 (0.01)	0.48 (0.01)
High school/above	0.09 (0.02)	0.09 (0.01)	0.08 (0.01)	0.08 (0.01)	0.20 (0.03)	0.20 (0.01)	0.13 (0.01)	0.15 (0.01) ***
Income (Low)	0.31 (0.03)	0.45 (0.02)	0.34 (0.01)	0.30 (0.01) ***	0.26 (0.03)	0.27 (0.02)	0.26 (0.01)	0.23 (0.01)
Income (Medium)	0.56 (0.03)	0.40 (0.02)	0.40 (0.01)	0.33 (0.01) ***	0.42 (0.03)	0.39 (0.02)	0.34 (0.01)	0.24 (0.01) ***
Income (High)	0.13 (0.02)	0.14 (0.01)	0.27 (0.01)	0.37 (0.01) ***	0.31 (0.02)	0.34 (0.02)	0.40 (0.01)	0.52 (0.01) ***
Married	0.94 (0.02)	0.93 (0.01)	0.93 (0.01)	0.93 (0.01)	0.89 (0.02)	0.91 (0.01)	0.89 (0.01)	0.90 (0.01)
Employed	0.78 (0.03)	0.68 (0.01)	0.69 (0.01)	0.69 (0.01) **	0.88 (0.02)	0.89 (0.01)	0.86 (0.01)	0.88 (0.01)
Smoking	0.02 (0.10)	0.06 (0.10)	0.04 (0.10)	0.04 (0.10) **	0.60 (0.03)	0.68 (0.02)	0.67 (0.01)	0.66 (0.01)
Drinking	0.08 (0.02)	0.08 (0.10)	0.09 (010)	0.08 (0.10)	0.69 (0.03)	0.71 (0.02)	0.70 (0.01)	0.69 (0.01)
Sedentary activity (h/w)	16.30 (1.13)	14.62 (0.41)	16.98 (0.30)	16.10 (0.28) ***	17.67 (1.01)	16.18 (0.49)	18.15 (0.35)	16.97 (0.32) ***
Total Calorie (1k kcal)	2.30 (0.04)	2.30 (0.02)	2.10 (0.01)	2.00 (0.01) ***	2.96 (0.06)	2.82 (0.03)	2.54 (0.02)	2.49 (0.02) ***
Fat (%E)	30.10 (0.72)	28.31 (0.35)	29.28 (0.22)	31.89 (0.26) ***	30.69 (0.77)	27.81 (0.36)	28.95 (0.22)	31.05 (0.28) ***
Carbohydrates (%E)	57.79 (0.74)	58.31 (0.40)	57.34 (0.23)	56.06 (0.25) ***	54.96 (0.85)	57.21 (0.45)	56.19 (0.26)	55.36 (0.29) ***
WC (cm)	79.75 (0.57)	80.19 (0.30)	81.33 (0.23)	82.69 (0.26) ***	83.26 (0.63)	82.67 (0.32)	83.36 (0.24)	84.81 (0.27) ***
BMI (kg/m^2^)	23.30 (0.20)	23.74 (0.11)	23.58 (0.08)	23.90 (0.09) **	23.05 (0.20)	23.22 (0.11)	23.29 (0.08)	23.66 (0.10) ***
General Obesity	0.07 (0.02)	0.12 (0.01)	0.11 (0.10)	0.13 (0.10) **	0.06 (0.02)	0.08 (0.01)	0.08 (0.01)	0.09 (0.01)
Abdominal Obesity	0.29 (0.03)	0.30 (0.01)	0.35 (0.01)	0.40 (0.01) ***	0.24 (0.03)	0.22 (0.01)	0.26 (0.01)	0.31 (0.01) ***
Sample Size	247	1042	2011	1947	229	924	1813	1632

Note: (1) The statistics reported are the sample mean with standard errors in parenthesis. (2) ***, **, and * indicate statistical significance at the 1%, 5%, and 10% level, respectively. (3) %E, share of calories; WC, waist circumstance; BMI, body-mass index.

**Table 3:** Effects of new rural cooperative medical insurance (NRCMS) on lifestyle behavior choices and risk of obesity.

Dependent Variable	Female			Male		
	Model 1	Model 2	Model 3	Model 1	Model 2	Model 3
*Panel A Obesity risk related indicators.*						
Total calorie (1k kcal)	0.03 (0.01) *		−0.01 (0.05)	0.11 (0.02) ***		0.09 (0.06)
Fat (%E)	−0.18 (0.27)		8.01 (0.90) ***	−0.18 (0.26)		7.35 (0.88) ***
Carbohydrates (%E)	0.65 (0.27) **		−9.00 (0.93) ***	0.47 (0.11)		−9.74 (0.98) ***
Sedentary activity (h/w)	−0.35 (0.34)		6.48 (1.11) ***	−1.89 (0.38) ***		5.47 (1.23) ***
Drinking		0.01 (0.05)	0.25 (0.12) **		0.09 (0.03) ***	−0.05 (0.11)
Smoking		0.15 (0.07) **	0.17 (0.23)		0.07 (0.03) **	0.25 (0.11) **
*Panel B Obesity indicators*						
WC (cm)	0.31 (0.24)		1.97 (0.79) **	−1.63 (0.25) ***		1.80 (0.82) **
BMI (kg/m^2^)	0.03 (0.24)		0.58 (0.28) **	−0.51 (0.09) ***		0.20 (0.47)
General Obesity		0.05 (0.04)	0.53 (0.14) ***		−0.18 (0.18) ***	−0.06 (0.16)
Abdominal Obesity		0.06 (0.04) *	0.30 (0.11) ***		−0.16 (0.04) ***	0.27 (0.12) **

Note: (1) The statistics reported are the marginal effects of independent variables with standard errors in parenthesis. (2) ***, **, and * indicate statistical significance at the 1%, 5%, and 10% level, respectively. (2) Control variables include: age, education levels, income levels, work status, employment status, marriage status, province dummies, and survey year dummies. (3) WC, waist circumstance; %E, share of calories. (4) Model 1: Ordinary least squares estimation (OLS) is used for regressions on total calories, fat (%E), carbohydrate (%E), sedentary activity, WC, and BMI. Model 2: the probit model is used for drinking, smoking, general obesity, and abdominal obesity. Model 3: model 1/model2 with an additional instrumental variable (IV).

**Table 4:** Effects of NRCMS on the probability of having general obesity and abdominal obesity.

Dependent Variable		Female			Male	
	Model 4	Model 5	Model 6	Model 4	Model 5	Model 6
WC (cm)	0.34 (0.17)		1.59 (0.78) **	−1.62 (0.25) ***		1.09 (0.80)
BMI (kg/m^2^)	0.04 (0.09)		0.47 (0.30) **	−0.51 (0.27) ***		0.14 (0.27)
General Obesity		0.04 (0.04)	0.51 (0.14) ***		−0.18 (0.05) ***	−0.09 (0.15)
Abdominal Obesity		0.07 (0.04)	0.24 (0.11) **		−0.11 (0.04) ***	0.20 (0.11) ^**^

Note: (1) The statistics reported are the marginal effects of independent variables with standard errors in parenthesis. (2) ***, **, and * indicate statistical significance at the 1%, 5%, and 10% level, respectively. (2) Control variables include: age, education levels, income levels, employment status, marriage status, sedentary activity time, daily calorie intake, the share of calories from carbohydrate and the share of calories from fat, drinking, smoking, and province dummies and survey year dummies; (3) WC, waist circumstance; %E, share of calories. (4) Model 4: Ordinary least squares estimation (OLS) is used for regressions on WC and BMI. Model 5: the probit model is used for general obesity and abdominal obesity. Model 6: model 4/model5 with an additional instrumental variable (IV).

## Appendix A

**Table A1:** Baseline characteristics of 2004–2011 CHNS population by health insurance status.

Characteristic	Total	Uninsured	Insured
*Demographic Characteristics*			
Age (year)	44.60 (11.90)	43.86 (11.81)	45.36 (11.90)
Female	0.53 (0.50)	0.52 (0.50)	0.53 (0.50) *
*Socioeconomic Status*			
Education level			
Primary/illiterate	0.42 (0.49)	0.35 (0.48)	0.48 (0.50) ***
Junior	0.38 (0.49)	0.36 (0.48)	0.40 (0.49) ***
High school/above	0.20 (0.40)	0.29 (0.45)	0.12 (0.32) ***
Income level			
Low	0.34 (0.47)	0.37 (0.48)	0.30 (0.46) ***
Medium	0.34 (0.47)	0.33 (0.47)	0.36 (0.48) ***
High	0.32 (0.47)	0.30 (0.46)	0.35 (0.48) ***
Married	0.90 (0.30)	0.89 (0.32)	0.91 (0.28) ***
Employed	0.74 (0.44)	0.71 (0.46)	0.78 (0.42) ***
*Lifestyle Behavior Choice*			
Smoking	0.33 (0.47)	0.33 (0.47)	0.34 (0.47) ***
Drinking	0.36 (0.48)	0.35 (0.48)	0.37 (0.48) ***
Sedentary activity (h/w)	17.30 (14.41)	0.18 (0.15)	0.17 (0.14) ***
Total Calorie (1k kcal)	2.32 (0.72)	2.30 (0.70)	2.34 (0.74) ***
Fat (%E)	29.0 (11.1)	28.13 (11.45)	29.84 (10.74) ***
Carbohydrates (%E)	57.50 (12.00)	58.38 (12.28)	56.59 (11.65) ***
*Obesity indicators*			
WC (cm)	81.97 (10.30)	81.36 (10.11)	82.57 (10.45) **
BMI (kg/m^2^)	23.50 (3.63)	23.41 (3.48)	23.57 (3.63) **
General Obesity	0.10 (0.29)	0.09 (0.29)	0.10 (0.30) ***
Abdominal Obesity	0.29 (0.45)	0.26 (0.44)	0.31 (0.46) ***
Sample Size	19577	9372	9845

Notes: (1) Date comes from CHNS longitudinal data, 2004, 2006, 2009, and 2011. (2) The statistics reported are the sample mean with the standard deviation in parentheses. (3) %E, share of calories; WC, waist circumstance; (4) ***, **, and * indicate statistical significance at the 1%, 5%, and 10% level, respectively.

**Table A2:** Basic characteristics trends of NRCMS participation from 2004 to 2011.

Variable	2004	2006	2009	2011
*Demographic Characteristics*				
Age (year)	44.38 (0.52)	45.25 (0.25)	44.58 (0.20)	46.37 (0.20) ***
Female	0.52 (0.02)	0.53 (0.01)	0.53 (0.01)	0.54 (0.01)
*Socioeconomic Status*				
Education level				
Primary/illiterate	0.50 (0.02)	0.48 (0.01)	0.48 (0.01)	0.48 (0.01)
Junior	0.37 (0.02)	0.37 (0.01)	0.41 (0.01)	0.41 (0.01) **
High school/above	0.14 (0.02)	0.14 (0.01)	0.11 (0.00)	0.12 (0.01) ***
Income level				
Low	0.29 (0.02)	0.37 (0.01)	0.30 (0.01)	0.27 (0.01) ***
Medium	0.50 (0.02)	0.40 (0.01)	0.37 (0.01)	0.29 (0.01) ***
High	0.22 (0.02)	0.24 (0.01)	0.33 (0.01)	0.44 (0.01) ***
Married	0.91 (0.01)	0.92 (0.01)	0.91 (0.00)	0.92 (0.00)
Employed	0.83 (0.02)	0.78 (0.01)	0.77 (0.01)	0.77 (0.01) **
*Lifestyle Behavior Choice*				
Smoking	0.30 (0.02)	0.35 (0.01)	0.34 (0.01)	0.32 (0.01) *
Drinking	0.38 (0.02)	0.38 (0.01)	0.38 (0.01)	0.36 (0.01)
Sedentary activity (h/w)	16.96 (0.77)	15.36 (0.32)	17.53 (0.23)	16.50 (0.21) ***
Total Calorie (1k kcal)	2.618 (0.04)	2.53 (0.02)	2.31 (0.01)	2.22 (0.12) ***
Fat (%E)	30.39 (0.53)	28.08 (0.25)	29.12 (0.16)	31.51 (0.19) ***
Carbohydrates (%E)	11.72 (0.11)	11.70 (0.05)	11.92 (0.04)	11.83 (0.04) ***
*Obesity-related indicator*				
WC (cm)	81.41(0.43)	81.34(0.22)	82.29 (0.17)	83.66(0.19) ***
BMI (kg/m^2^)	23.18(0.14)	23.50(0.08)	23.44 (0.06)	23.79(0.07) ***
General Obesity	0.06 (0.01)	0.10 (0.01)	0.09 (0.01)	0.11(0.01) ***
Abdominal Obesity	0.26 (0.02)	0.26 (0.01)	0.31 (0.01)	0.36(0.01) ***
Sample Size	476	1966	3824	3579

Notes: (1) Date comes from CHNS longitudinal data, 2004, 2006, 2009, and 2011. (2) The statistics reported are the sample mean with the standard errors in parentheses. (3) %E, share of calories; WC, waist circumstance; (4) ***, **, and * indicate statistical significance at the 1%, 5%, and 10% level, respectively.

**Table A3:** Effects of the NRCMS on a female's lifestyle choice and being obese.

Explanatory Variables	Lifestyle Choice						Obesity-Related Indicator			
	Calorie	Fat (%E)	Carbohydrate (%E)	Sedentary	Dinking	Smoking	BMI	WC	General	Abdominal
NRCMS	−7.21	8.01 ***	−9.00 ***	6.48 ***	0.63 ***	0.17	0.58 **	1.97 **	0.53 ***	0.16
	47.33	(0.90)	(0.93)	(1.11)	(4.35)	(0.23)	(0.28)	(0.79)	(0.14)	(0.11)
Age (year)	21.14 **	0.14 *	−-0.17 **	−0.44 ***	0.03 **	0.07 ***	0.27 ***	0.44 ***	0.05 ***	0.07 ***
	(3.78)	(0.07)	(0.07)	(0.09)	(2.48)	(0.24)	(0.02)	(0.06)	(0.01)	(0.01)
Junior	−28.34 *	2.64 ***	−3.00 ***	2.68 ***	0.10 **	−0.18 **	0.18 **	−0.37	0.05	−0.02
	(14.76)	(0.28)	(0.29)	(0.35)	(2.17)	(0.07)	(0.09)	(0.25)	(0.04)	(0.03)
High/above	−99.68 **	6.88 ***	−8.37 ***	8.10 ***	0.53 ***	−0.51 ***	−0.28 **	−1.34 ***	−0.01	−0.13 **
	(23.80)	(0.45)	(0.47)	(0.56)	−7.74	(013)	(0.14)	(0.40)	(0.07)	(0.05)
Income (Medium)	34.08 **	0.35	−0.18	−0.40	0.02	−0.08	−0.00	−0.59 **	−0.05	−0.06 *
	(14.56)	(0.28)	(0.29)	(0.34)	(0.47)	(0.07)	(0.09)	(0.24)	(0.04)	(0.03)
Income (High)	19.38	2.70 ***	−2.86 ***	0.71 *	0.18 ***	−0.15 *	0.06	−0.25	0.03	−0.05
	(17.33)	(0.33)	(0.34)	(0.41)	(3.40)	(0.09)	(0.10)	(0.29)	(0.05)	(0.04)
Married	71.74 ***	−1.19 ***	0.93 **	−0.84 *	−0.17 ***	−0.13	0.03	0.47	−0.06	0.08 *
	(21.45)	(0.41)	(0.42)	(0.50)	(−2.60)	(0.10)	(0.19)	(0.35)	(0.07)	(0.05)
Employed	57.36 ***	−2.56 ***	2.72 ***	−2.08 ***	0.02	−0.09	−0.48 ***	−1.45 ***	−0.18 ***	−0.17 ***
	(14.42)	(0.27)	(0.28)	(0.34)	(0.42)	(0.07)	(0.09)	(0.24)	(0.04)	(0.03)
Year dummies	yes	yes	yes	yes	yes	yes	yes	yes	yes	yes
Province dummies	yes	yes	yes	yes	yes	yes	yes	yes	yes	yes
Hausman test *p*-value	0.04	0.00	0.00	0.00	0.00	0.00	0.00	0.00	0.00	0.00
First-stage F	913.92	913.92	913.92	908.97	913.92	913.92	913.92	866.64	913.92	913.92
Sample Size	10,305	10,305	10,305	10,305	10,305	10,305	10,305	10,305	10,305	10,305

Notes: (1) Date comes from CHNS longitudinal data, 2004, 2006, 2009, and 2011. (2) The statistics reported are the sample mean with the standard errors in parentheses. (3) %E, share of calories; WC, waist circumstance; (4) ***, **, and * indicate statistical significance at the 1%, 5%, and 10% level, respectively.

**Table A4:** Effects of the NRCMS on a male's lifestyle behavior choice and being obese.

Explanatory Variables	Lifestyle Choice						Obesity-related Indicator			
	Calorie	Fat (%E)	Carbohydrate (%E)	Sedentary	Dinking	Smoking	BMI	WC	General	Abdominal
NRCMS	87.82	−7.36 ***	−9.74 ***	5.47 ***	0.25 **	−0.05	0.20	1.80 **	−0.06	0.29 ***
	(61.12)	(0.88)	(0.97)	(1.23)	(0.10)	(0.11)	(0.28)	(0.82)	(0.16)	(0.11)
Age (year)	21.80 ***	0.20 ***	−0.33 ***	−0.81 ***	0.05 ***	0.05 ***	0.16 ***	0.49 ***	0.03 ***	0.05 ***
	(4.71)	(0.07)	(0.08)	(0.10)	(0.01)	(0.01)	(0.02)	(0.06)	(0.01)	(0.01)
Junior	−20.52	1.75 ***	−1.98 ***	1.98 ***	0.02	−0.02	0.53 ***	1.67 ***	0.21 ***	0.22 **
	(19.04)	(−0.27)	(0.31)	(0.38)	(0.03)	(0.03)	(0.09)	(0.25)	(0.05)	(0.03)
High/above	−96.04 ***	5.24 ***	−6.22 ***	7.97 ***	0.13 ***	−0.16 ***	1.06 ***	3.15 ***	0.02 ***	0.44 ***
	(26.86)	(0.39)	(0.43)	(0.54)	(0.05)	(0.05)	(0.12)	(0.36)	(0.05)	(0.05)
Income (Medium)	78.05 ***	0.25	−0.04	−0.30	0.11 ***	0.04	−0.18 *	−0.61 **	−0.06	−0.10 ***
	(20.92)	(0.30)	(0.33)	(−0.42)	0.04	(0.04)	(0.09)	(0.28)	(0.05)	(0.04)
Income (High)	56.17 **	2.73 ***	−3.60 ***	1.13 **	0.17 ***	0.02	0.25 **	0.76 ***	0.02	0.08 **
	(22.00)	(0.32)	(0.36)	(0.44)	(0.04)	(0.04)	(0.10)	(0.29)	(0.05)	(0.04)
Married	−1.17	0.72	−0.67	−1.88 ***	0.20 ***	0.10 **	0.66 ***	1.60 ***	0.25 ***	0.17 ***
	(26.69)	(0.39)	(0.43)	(0.54)	(0.05)	(0.05)	(0.12)	(0.35)	(0.08)	(0.05)
Employed	111.52 ***	−2.76 ***	2.90 ***	−4.25 ***	0.15 ***	0.16 ***	−0.35 ***	−1.72 ***	−0.13 **	−0.22 ***
	(24.30)	(0.35)	(0.39)	(0.49)	(0.04)	(0.04)	(0.11)	(0.32)	(0.06)	(0.04)
Year dummies	yes	yes	yes	yes	yes	yes	yes	yes	yes	yes
Province dummies	yes	yes	yes	yes	yes	yes	yes	yes	yes	yes
Hausman test *p*-value	0.00	0.00	0.00	0.00	0.00	0.00	0.00	0.00	0.00	0.00
First−stage F	731.04	731.04	731.04	728.80	731.04	731.04	731.04	683.89	731.04	731.04
Sample Size	9272	9272	9272	9272	9272	9272	9272	9272	9272	9272

Notes: (1) Date comes from CHNS longitudinal data, 2004, 2006, 2009, and 2011. (2) The statistics reported are the sample mean with the standard errors in parentheses. (3) %E, share of calories; WC, waist circumstance; (4) ***, **, and * indicate statistical significance at the 1%, 5%, and 10% level, respectively.

**Table A5:** Effects of the NRCMS on the probabilities of having general obesity and abdominal obesity.

Explanatory Variables	Female				Male			
	BMI	WC	Obesity	Abdominal	BMI	WC	Obesity	Abdominal
NRCMS	0.47 (0.28) *	1.59 (0.78) **	0.51 (0.13) ***	0.12 (0.11)	0.06 (0.27)	1.08 (0.80)	−0.09 (0.15)	0.20 (0.10) **
Age (year)	0.27 (0.02) ***	0.44 (0.06) ***	0.04 (0.01) **	0.07 (0.01) ***	0.16 (0.02) ***	0.49 (0.06) ***	0.03 (0.01) ***	0.05 (0.01) ***
Junior	0.12 (0.09)	−0.52 (0.25) **	0.04 (0.04)	−0.03 (0.03)	0.49(0.09) ***	1.52 (0.25) ***	0.20 (0.05) ***	0.20 (0.03) ***
High school/above	−0.43 (0.14) ***	−1.76 (0.40) ***	−0.04 (0.07)	−0.16 (0.05) ***	0.93 (0.12) **	2.61 (0.35) ***	0.22 (0.07) ***	0.37 (0.05) ***
Income (Medium)	−0.02 (0.09)	−0.62 (0.24) **	−0.06 (0.07)	−0.06 (0.03) *	−0.19 (0.09) **	−0.64 (.27) ***	0.03 (0.08) ***	−0.11 (0.04) ***
Income (High)	0.02 (0.10)	−0.38 (0.29)	−0.05 (0.04)	−0.06 (0.04)	0.20 (0.09) **	0.50 (0.29) **	−0.07 (0.06)	0.05 (0.04)
Married	0.02 (0.13)	0.42 (0.36)	0.02 (0.05)	0.08 (0.05)	0.67 (0.12) ***	1.55 (0.35) *	0.01 (0.06)	0.17 (0.05) ***
Employed	−0.44 (0.09) ***	−1.34 (0.24) ***	−0.16 (0.04) ***	−0.16 (0.03)	−0.28 (0.11) ***	−1.47 (0.32) ***	−0.12 (0.06) **	−0.18 (0.04) ***
Drinking	−0.08 (0.13)	−0.27 (0.35)	−0.08 (0.07)	−0.06 (0.05)	0.15 (0.08)	0.78 (0.23) ***	0.02 (0.04)	0.06 (0.03) **
Smoking	−0.62 (0.19) ***	−1.36 (0.53) **	0.01 (0.00)	−0.28 (0.08) ***	−0.51 (0.07) ***	−0.99 (0.22) ***	−0.15 (0.04) ***	−0.12 (0.03) ***
Sedentary	0.01 (0.01) *	0.01 (0.01)	0.01 (0.00)	0.01 (0.01)	0.01 (0.00)	0.01 (0.01)	−0.01 (0.01)	0.01 (0.01)
Total calorie	0.00 (0.00)	0.00 (0.00)	0.00 (0.00)	0.00 (0.00)	0.00 (0.00)	−0.00 (0.00)	0.01 (0.01)	0.00 (0.00)
Fat (%E)	−0.00 (0.01)	−0.03 (0.02)	0.01 (0.01)	−0.01 (0.00)	0.02 (0.01) ***	0.06 (0.02)	0.01 (0.01)	0.01 (0.00) **
Carbohydrate (%E)	−0.010 (0.01) *	−0.06 (0.02) ***	−0.01 (0.01)	−0.01 (0.00)	0.00 (0.01)	−0.01 (0.02) ***	0.01 (0.01)	−0.01 (0.00)
Year dummies	yes	yes	yes	yes	yes	yes	yes	yes
Province dummies	yes	yes	yes	yes	yes	yes	yes	yes
Hausman test *p*-value	0.00	0.00	0.00	0.00	0.00	0.00	0.00	0.00
First-stage F	714.06	714.06	714.06	714.06	570.51	570.51	570.51	570.51
Sample Size	10,278	10,278	10,278	10,278	9255	9255	9255	9255

Notes: (1) Date comes from CHNS longitudinal data, 2004, 2006, 2009, and 2011. (2) The statistics reported are the sample mean with the standard errors in parentheses. (3) %E, share of calories; WC, waist circumstance; (4) ***, **, and * indicate statistical significance at the 1%, 5%, and 10% level, respectively.
